# Regulation of Transcriptional Bursting by a Naturally Oscillating Signal

**DOI:** 10.1016/j.cub.2013.12.011

**Published:** 2014-01-20

**Authors:** Adam M. Corrigan, Jonathan R. Chubb

**Affiliations:** 1 MRC Laboratory for Molecular Cell Biology and Department of Cell and Developmental Biology, University College London, Gower Street, London WC1E 6BT, UK

## Abstract

Transcription is highly stochastic, occurring in irregular bursts [1, 2, 3]. For temporal and spatial precision of gene expression, cells must somehow deal with this noisy behavior. To address how this is achieved, we investigated how transcriptional bursting is entrained by a naturally oscillating signal, by direct measurement of transcription together with signal dynamics in living cells. We identify a *Dictyostelium* gene showing rapid transcriptional oscillations with the same period as extracellular cAMP signaling waves. Bursting approaches antiphase to cAMP waves, with accelerating transcription cycles during differentiation. Although coupling between signal and transcription oscillations was clear at the population level, single-cell transcriptional bursts retained considerable heterogeneity, indicating that transcription is not governed solely by signaling frequency. Previous studies implied that burst heterogeneity reflects distinct chromatin states [4, 5, 6]. Here we show that heterogeneity is determined by multiple intrinsic and extrinsic cues and is maintained by a transcriptional persistence. Unusually for a persistent transcriptional behavior, the lifetime was only 20 min, with rapid randomization of transcriptional state by the response to oscillatory signaling. Linking transcription to rapid signaling oscillations allows reduction of gene expression heterogeneity by temporal averaging, providing a mechanism to generate precision in cell choices during development.

## Highlights

• We observe coupled signal and transcriptional oscillations in living cells
• Oscillations are collectively robust but heterogeneous at the single-cell level
• Response heterogeneity is maintained by a short-term transcriptional persistence
• Oscillations can be a mechanism to control gene expression noise in development

## Teaser

Corrigan and Chubb, through live imaging of transcriptional bursts, observe coupled oscillations between an extracellular signal and downstream transcription. Oscillations provide a mechanism to reduce transcriptional noise.

## Results and Discussion

### Oscillatory Transcription of the *csaA* Gene

Transcription occurs in irregular bursts or pulses. Despite this noisy behavior, organisms manage to achieve spatial and temporal precision in their gene expression. A growing body of evidence indicates that the temporal dynamics of signaling can provide specificity in transcription [7, 8, 9, 10, 11, 12] in a diverse range of contexts, from the DNA damage response to calcium, ERK, NF-κB, insulin, and steroid signaling. Information coding by signaling dynamics has been proposed to offer several advantages over signal amplitude in the regulation of gene expression [7, 8, 13, 14]. However, it is unclear how signaling regulates the noise inherent in the transcriptional process to generate precision in gene expression. Previous studies have measured effects of signal dynamics by relating exogenous stimulation to reporter protein expression or steady-state population RNA levels. However, to understand how accurate expression emerges from noisy transcription requires direct visualization of the dynamics of the actual bursts, without the temporal or population averaging of standard gene expression measures. In addition, although exogenous stimulation is a useful approach, to understand transcriptional regulation in natural biological contexts it is necessary to investigate natural signaling dynamics. Here we investigated how oscillatory signaling entrains transcription by direct imaging of transcriptional bursts, in combination with measurements of the natural dynamics of the transcriptional inducer. The period of oscillation is on the timescale of minutes, considerably faster than previously observed gene expression oscillations. By tracking cells over successive oscillations, we measure the timescale of transcriptional variability between cells and show that oscillating stimulation is a mechanism that can act to override variability and generate accuracy in gene expression.

To visualize transcriptional bursts, we inserted MS2 repeats [15] into a gene and detected them using the MS2-GFP protein, which has a high affinity, sequence-specific interaction with MS2 RNA stem loops, allowing RNA appearance at the transcription site to be visualized as a fluorescent spot. Most genes show exponentially distributed burst durations [16]. The *Dictyostelium csaA* gene, which encodes a developmentally induced cell adhesion glycoprotein [17], does not show robust exponential bursts [16]. Closer examination of *csaA* bursting data revealed periodic fluctuations in spot intensity (see Figure S1A available online) indicative of oscillatory transcription.

*csaA* is induced by extracellular cAMP [18, 19, 20]. During early development, cAMP signaling between cells causes cell aggregation. Cell responses of signal amplification and transient desensitization cause oscillatory waves of cAMP to propagate throughout the population [21, 22, 23, 24, 25, 26]. Waves are first detected at 3–4 hr of development, before a progressive increase in oscillation frequency as cells form streams, then mounds. To understand the mechanisms controlling *csaA* oscillations, we used high-content imaging to analyze *csaA* transcription at multiple times between differentiation onset (0 hr) and cell streams (6 hr).

**Figure 1:**
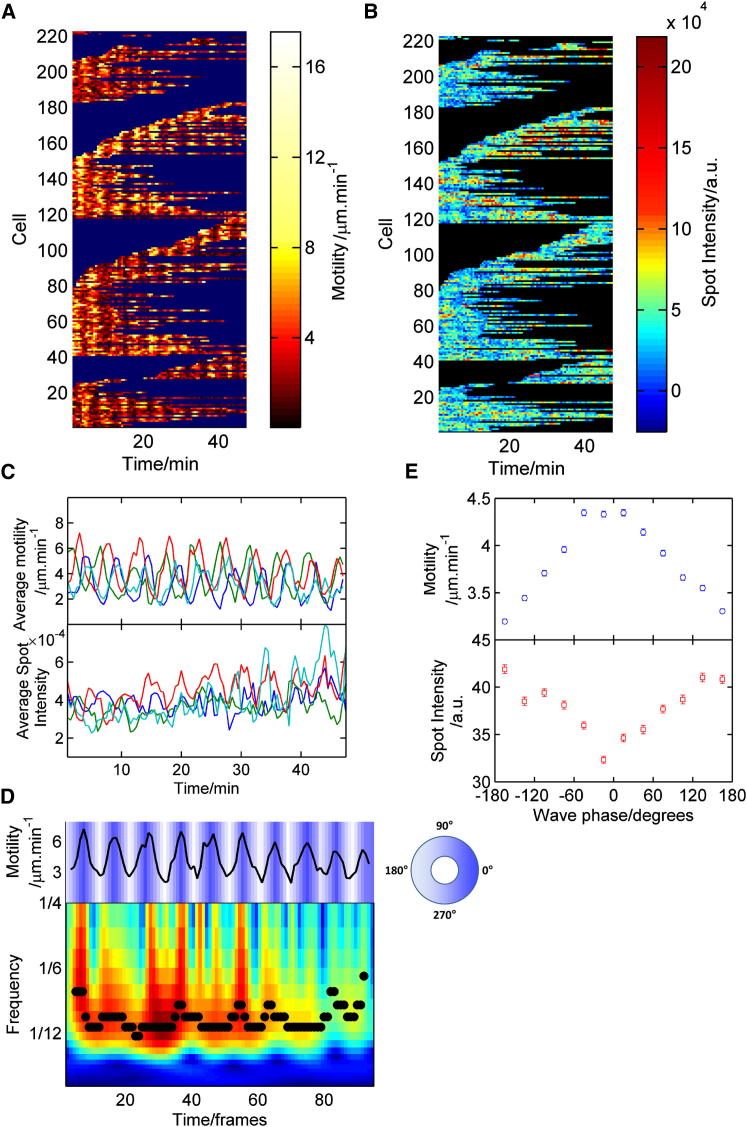
Oscillations in Transcription and Motility (A) Two-frame displacement for individual cells tracked over time for four fields of view at 5 hr development. Each row represents one cell. Color denotes two-frame motility (μm/min; black [low] to white [high]). Dark areas indicate where a cell has not been tracked. Data from four stage positions were captured simultaneously. Cell tracks are organized by track starting time during capture. (B) Transcription spot intensity in the same cells as in (A). (C) Displacement (upper) and spot intensity (lower) averaged over each field of view reveal clear oscillations. (D) Wavelet analysis: the averaged displacement for a single field of view (top panel, solid line). Phase is indicated by background color. Bottom panel: wavelet transform of the displacement data; ridge points are indicated by black circles. (E) Displacement (top panel) and transcription (bottom panel) data grouped by motility phase. The phase lag was estimated by fitting a sinusoidal function to the cross-correlation between motility and transcription. Error bars indicate SEM.

Cell speed (measured as the two-frame displacement) was used as a proxy for cAMP wave phase [23]. Clear vertical striations are visible in motility traces (Figure 1A), indicating that cells across several fields of view exhibited synchronous bursts of motility. In equivalent plots of transcription spot intensity (Figure 1B), collective oscillations are less apparent, with considerable cell heterogeneity. Since waves of cAMP were synchronous across fields of view, we reduced noise by averaging cell behavior in each frame, revealing robust oscillatory behavior in each field for both motility (Figure 1C, top) and transcription (Figure 1C, bottom).

Autocorrelation of averaged motility (Figure S1F, top left) reveals a peak around 5 min, the wave period of motility at 5 hr. Similarly, average transcription intensity autocorrelations (Figure S1F, top right) peak with the same lag, indicating that motility and transcription oscillations have the same frequency. Waves were also apparent in the displacement autocorrelation for single cells (Figure S1F, lower left). The single-cell transcription autocorrelation displayed very weak periodicity (Figure S1F, lower right), indicating a temporally heterogeneous response. There was a weak correlation between motility and transcriptional output of a cell within a motility cycle (Figure S1G), suggesting a common sensing mechanism. The correlation disappeared during cell aggregation, perhaps reflecting geometric constraints on motility.

### Phase Shift between Signaling and Transcription Waves

**Figure 2:**
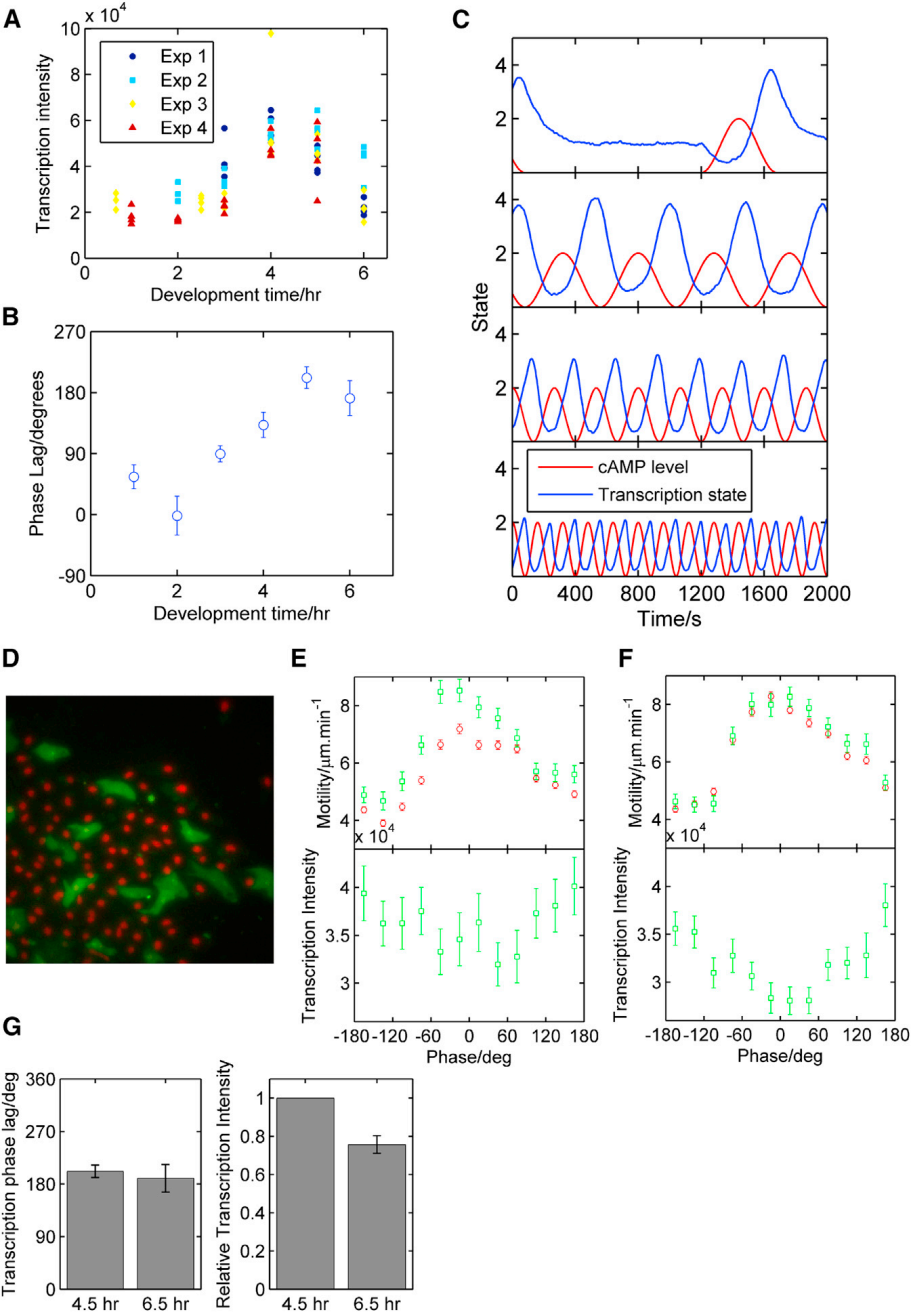
Developmental Changes in Transcriptional Strength and Phasing (A) Variation of transcription intensity with developmental time. Each data point represents a field of view. Colors denote distinct experiments. (B) Phase lag between motile and transcriptional responses. The circular mean and circular SEM are displayed as a function of developmental time. (C) A simple model of transcriptional phasing with examples of average transcription state for a range of cAMP frequencies. Increasing cAMP wave frequency reduces transcription amplitude, in line with real data. (D) Cell mixing experiments address relative effects of cAMP wave timing and developmental time. (E) Representative experiments comparing motility and transcriptional phasing of 4.5 hr *csaA*MS2 cells (green) mixed with 6.5 hr cells (red). Four of six experimental repeats showed clear transcriptional oscillations (one of the two transcriptional nonoscillators showed no motility oscillation). (F) Comparison of motility and transcriptional phasing of 6.5 hr *csaA*MS2 cells (green) mixed with 6.5 hr cells (red). Five of six repeats showed clear transcriptional oscillations, with the nonoscillating repeat showing no motility periodicity. (G) Transcription phase lag (left) and relative spot intensity (right) for *csaA*MS2 cells in 4.5 hr and 6.5 hr mixes. Error bars indicate SEM.

Peaks of transcription spot intensity were not in synchrony with motility peaks (Figure 1C), with a clear phase shift between the two waves. To quantify transcription phase behavior, we used wavelet analysis to extract the phase of the motility wave (Figure 1D) [27]. The average transcription spot intensity varied depending on the motility phase (Figure 1E), with a mean lag after the motility peak of 162° (SD 39°; four fields of view). To define the developmental regulation of the oscillations, we imaged multiple time windows between 0 and 6 hr, over multiple experimental days. The average field-of-view spot intensity peaked around 4 hr, before decreasing by 6 hr (Figure 2A). The phase lag between motility and transcription also varied during differentiation (Figure 2B). At early times, the lag was small, around 90°. After wave onset, the phase lag increased to around 180° and remained close to antiphase in cell streams (6 hr). This plot trajectory provides in vivo support for a two-phase process of transcriptional regulation during early differentiation [18].

Why do cells show decreased spot intensity as cells begin aggregation? A previous study on NF-κB oscillations also observed a dampening effect as the frequency of stimulation increased [9], indicating that this system does not fully reset at higher frequencies. NF-κB models may not apply here, as NF-κB repression relies on negative feedback from a target gene, whereas cAMP is a robustly oscillating external signal and so does not need an intracellular circuit to generate oscillations. As cAMP waves became faster upon aggregation, transcription remained approximately antiphase with the motility cycle, rather than encroaching on the next motility wave, ruling out a simple time delay as the sole regulator of the transcriptional lag. Together with the high-frequency repression, this suggested a model with stimulation and repression at different points in the signal wave. We constructed a very simple model for *csaA* regulation, wherein a cell has a number of activity states of monotonically increasing initiation rate (Figure S2A); transition rates between states depend on the cAMP gradient such that downward transitions are enhanced during the rising phase of the cAMP wave and repressed during the falling phase (Figure S2B). We assumed rate constants independent of developmental time to determine whether cAMP dynamics alone can describe observed transcriptional profiles. An extended discussion of modeling considerations, including alternative model architectures, is contained in the Supplemental Results. The simulated average transcriptional activity (Figure S2C) showed qualitative agreement with experimental data (Figure 2A), increasing and then decreasing in intensity as wave frequency increased. The simulated phase lag between cAMP and transcription also increased at higher wave frequencies (Figure S2D), as observed when cells proceed through development (Figure 2B). Figure 2C illustrates the average transcriptional response for a simulated population of 1,000 cells to increasing cAMP wave frequency. Before robust cAMP waves (Figure 2C, top), the transcriptional response is strong, but transcription is less frequent, so the overall average is lower (0–3 hr in in Figure 2A). The transcription intensity remains high and approaches antiphase with the cAMP when waves become robust (4 hr; Figure 2C, second panel). Transcription declines as waves become closer together, turning off the gene before it becomes maximally active (Figure 2C, bottom two panels). This simple model illustrates the minimal elements required to reproduce observed intensity and timing behavior of transcription bursts. Although it is known that oscillation frequency can manifest spatially, for example by determining somite length in vertebrate embryos [28, 29], this model temporally links the onset, peak, and then repression of transcription to signal frequency, providing a timing mechanism to induce gene expression at the correct point in development.

To test the validity of the model, we generated cell mixes of 4.5 hr *csaA*MS2 cells mixed at a 20:80 ratio with 6.5 hr cells expressing only a nuclear marker. Control mixes used 6.5 hr *csaA*MS2 cells and 6.5 hr nuclear marker cells. If cAMP oscillation frequency is the sole driver of transcriptional behavior, we would expect 4.5 hr cells to respond to the faster waves of cAMP with the same periodicity and strength as 6.5 hr cells. The 4.5 hr cells entered streams (Figure 2D), and in four of five experimental repeats displaying motility oscillations, cells also showed robust transcriptional oscillations (Figure 2E). The timing of these oscillations was similar to 6.5 hr cells in control mixes (Figure 2F), in support of the model. However, for all experimental repeats, the average spot intensity was higher in 4.5 hr mixes than in 6.5 hr mixes (Figure 2G), as expected for 4–5 hr cells in standard conditions (Figure 2A). Therefore, although cAMP frequency can explain transcription oscillation dynamics, the strength and penetrance of the transcriptional response is additionally determined by intrinsic factors responsive to developmental time.

### Dynamics of Transcriptional Heterogeneity

The oscillations of signaling and transcription show clear coupling at the population level. However, transcription oscillations were discernible only in field-of-view averages and showed considerable noise at the single-cell level (Figure S3A). Some cells had a strong transcription spot throughout a trough, whereas others showed a weak spot in peaks. What are the sources of this variability in response to signal oscillations? Are they extrinsic or intrinsic to the cell?

**Figure 3:**
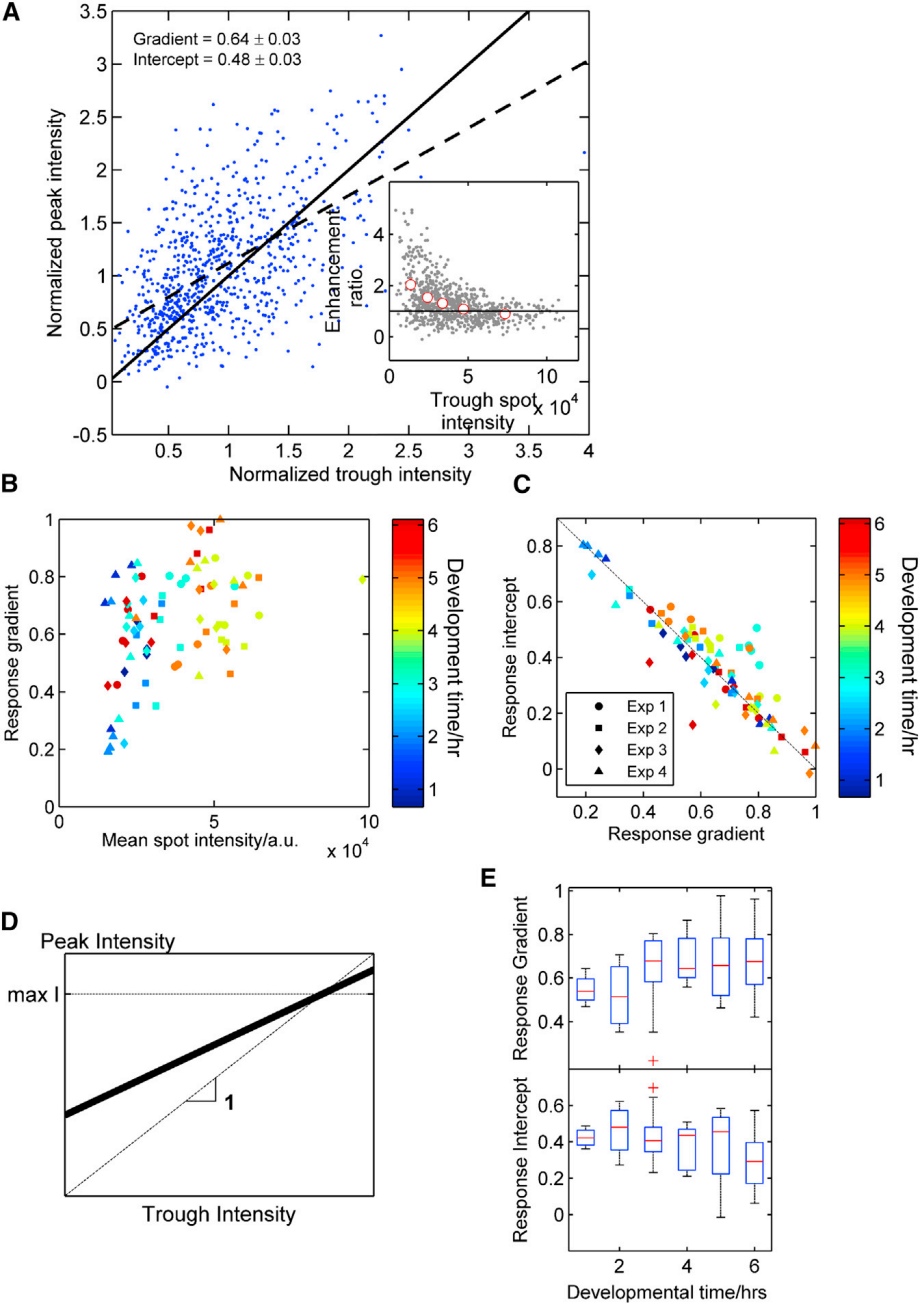
Quantifying Extrinsic and Intrinsic Regulation of the Transcriptional Response (A) Comparison of trough and subsequent peak transcription responses for a characteristic 5 hr field, normalized by field-of-view overall mean. The dotted line shows a linear fit to the data points with gradient and intercept indicated. The solid line indicates equal peak and trough intensities. Inset: the ratio of peak to trough response as a function of trough transcription intensity. (B) Relationship between response gradient and the mean spot intensity of fields of view. The measured correlation coefficient is 0.43 (p = 10^−4^). (C) Relationship between response gradient and intercept. (D) Cartoon illustrating the link between the response gradient and the balance between intrinsic and extrinsic variation. The thick black line shows a typical relationship between peak and trough intensities, intermediate between a line of gradient 1 (no response to cAMP) and a horizontal line (response determined entirely by cAMP). Alternative scenarios not supported by the data are described in Figure S3C. The scenario depicted in the cartoon is a consequence of the different forms of our wave model (Figures S3D and S3E). (E) Distribution of response gradient and intercept as a function of developmental time. Whiskers extend to the most extreme data point in the distribution not flagged as an outlier. Outliers fall more than 1.5-fold outside the interquartile range beyond the upper or lower quartile.

Most analyses measure only instantaneous cell-cell variability [6]; however, our approach allows us to measure how variability between cells changes over time. To quantitatively investigate the dynamics of this heterogeneity, we defined the transcriptional response of a cell to cAMP by measuring average spot intensities in troughs and subsequent peaks (Figures 3A and S3B). This allows us to understand how the response of the cell is determined. Simply put, if all cells have the same peak transcription independent of trough intensity, then we infer that the peak level is externally controlled, whereas if peak and trough intensities are similar, then the internal state of the cell is more important than the external signal. We observed strong correlation between trough and peak intensities (Figure 3A). As expected, the peak intensity tended to be greater than the preceding trough intensity (most points lie above y = x). A linear fit to the data has a gradient of 0.64 ± 0.03 (intercept 0.48 ± 0.03). A gradient < 1 means that as trough intensity becomes larger, the response becomes smaller, to a point where cells already transcribing strongly were unaffected by cAMP waves (Figure 3A inset, approximated by the intersection between the fit and y = x in Figure 3A), implying saturation of the transcription site. The response gradient correlated with the average field-of-view spot intensity (Figure 3B), again indicating an upper limit on transcript load.

We observed an inverse correlation between response gradient and intercept (Figure 3C), and together with the evidence of an upper limit on transcript load, this allows us to interpret the response profile as the balance between intrinsic and extracellular influences on transcription (Figure 3D; alternative theoretical models not fitting the data are treated in Figure S3C). A horizontal response means that the peak response is constant and does not depend on transcription intensity in the trough, and is instead determined by the cAMP wave. In contrast, a gradient of 1 (and zero intercept; Figure 3C) implies that peak and trough intensities are equal and that transcription is unaffected by the wave. Therefore, the response gradient scales with the relative importance of intrinsic factors, and the intercept estimates the fraction of transcriptional behavior determined by extracellular variations. Although changes in intercept and gradient occur during differentiation (Figure 3E), these changes are modest, implying that relative contributions of extrinsic and intrinsic regulation do not undergo large alterations despite substantial changes in cell physiology and responsiveness. However, as we demonstrate below, the properties of these response parameters allow insight into the regulation of heterogeneity.

### Regulation of Transcriptional Heterogeneity

**Figure 4:**
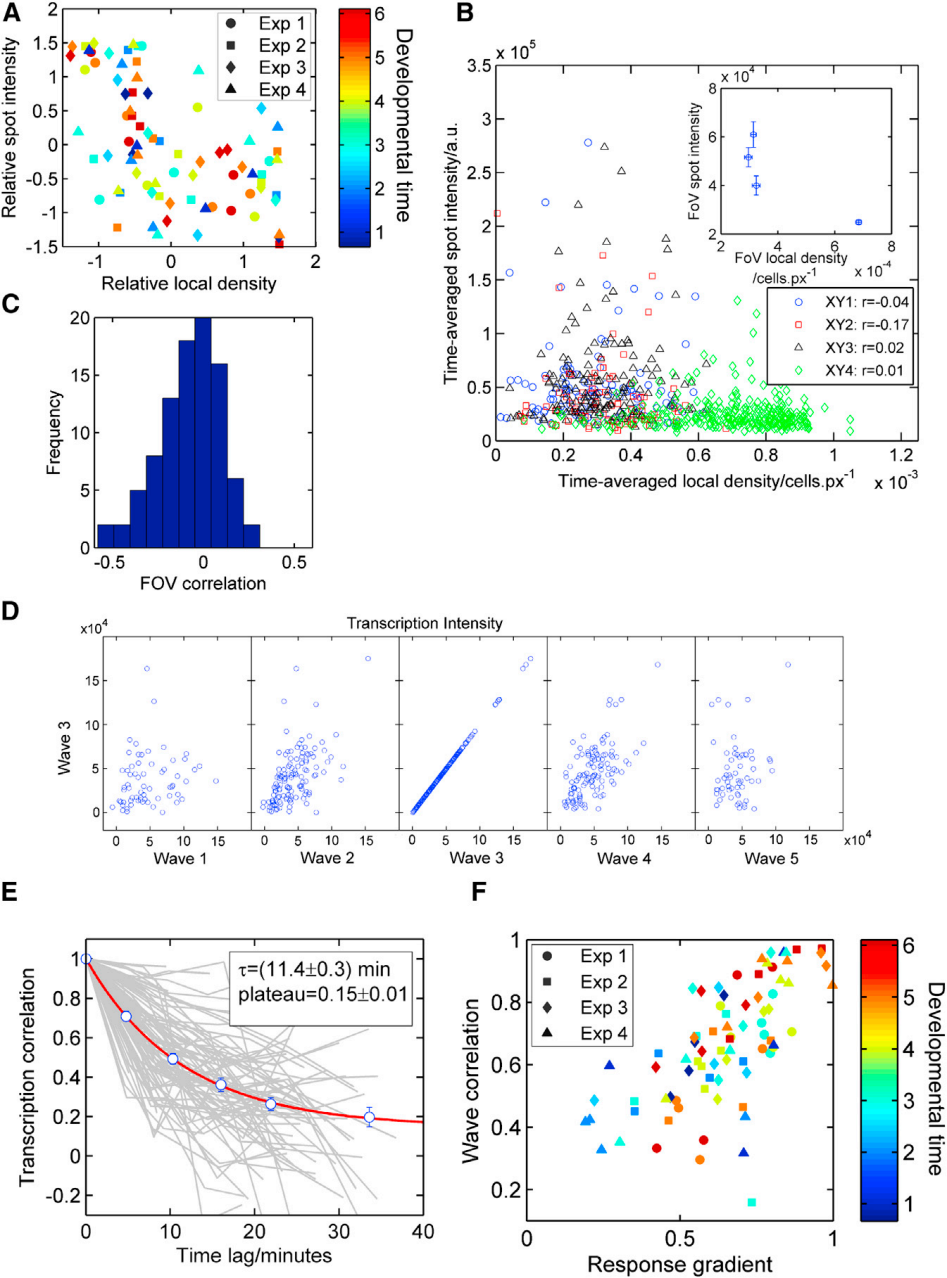
Sources of Transcriptional Heterogeneity (A) Fields of view more dense than average have weaker transcription than average. Each field is denoted by a marker, with color indicating developmental time and shape denoting imaging day. Points are normalized relative to other fields captured simultaneously. (B) Average spot intensity against average local density at 5 hr. Different markers represent four different fields of view captured simultaneously. A weak negative correlation is observed both within fields of view (main figure) and between fields (inset). Error bars indicate SEM. (C) Histogram of the weak and heterogeneous negative correlation within individual fields of view for all data at all time points. (D) Transcriptional response of individual cells to the third wave of cAMP (from start of image capture) compared with their response to waves 1, 2, 3, 4, and 5. (E) Ensemble of decay curves for transcriptional persistence (light gray lines) as a function of temporal separation. The average behavior (circles) is described by exponential decay to a nonzero plateau. Error bars indicate SEM. (F) Correlation between transcriptional persistence and the response gradient.

What mechanisms generate heterogeneity of response to the cAMP wave? We have identified both cell-extrinsic and cell-intrinsic contributions. A clear extrinsic driver of transcriptional heterogeneity is cell density, with transcription inhibited at high cell densities. A negative correlation was observed between transcription and field-of-view density (Figures 4A and S4A). Cells in dense regions have weaker transcription than those in sparse areas at all developmental times. Figure 4B plots spot intensity against local density at 5 hr for individual cells within four fields of view captured simultaneously. Comparing within individual fields gives weak negative correlations with a relatively high degree of heterogeneity (Figure 4C). The weak effect observed *within* each field of view means that there is some local effect whereby transcription is controlled based on the environment around each cell; however, the stronger effect *between* fields of view (Figure 4B inset) implies that the length scale of signaling is greater than the typical cell-cell separation (tens of micrometers) but less than the interfield distance of 1800 ± 100 μm. This length scale suggests cAMP signal strength as a possible source of heterogeneity. The manner in which *Dictyostelium* self-organizes into aggregation centers means that there are many environments in which cells find themselves. A cell must nevertheless differentiate with the correct timing appropriate to its local environment, from isolation, through streams to mounds, requiring adequate sensing and adaptation mechanisms.

The lack of clear periodic behavior in transcription in single cells implied that the response in each cell changes with successive waves and is not determined entirely by the extracellular signal. We compared intensities in a wave to adjacent waves for individual cells (Figure 4D). The response to wave 3 was strongly related to the responses in directly adjacent waves, with cells having an above average intensity in wave 3 likely to have above average intensities in waves 2 and 4. Waves 1 and 5 were also related to wave 3; however, the effect was weaker. These data indicate persistence in transcriptional activity.

The lifetime of the correlation between waves was considerably shorter than for other examples of transcriptional persistence [30]. Due to the limited number of cells in each field, correlation decay curves are noisy, particularly for large time lags (Figure 4E; see also Figure S4B). However, the average decay curve (circles) fits an exponential function (red curve) approaching a nonzero plateau. This behavior is suggestive of two timescales of transcriptional persistence, one decaying over a period of 20 min and another persisting beyond the imaging timeframe. The effects of cell density and transcriptional persistence on *csaA* oscillations appear independent, as the two measures were uncorrelated (Figure S4C). Short-term transcriptional persistence is a key feature of our wave model (Figures S4E and S4F). Transient persistence would buffer cells against short-term environmental fluctuations.

Persistence was dependent on the properties of *csaA* transcription. Persistence between successive waves was weakly correlated with mean and SD of field spot intensity (r = 0.26 and r = 0.21, respectively). In other words, cells with brighter spots showed greater persistence, and in heterogeneous populations (high SD), persistence will appear greater because deviations from the field average (and therefore fluctuation times) are large. The strongest correlation for transcriptional persistence occurred with the response parameters (Figure 4F). The persistence of transcription intensity between waves was proportional to the response gradient; put simply, the memory of transcriptional activity was lost faster in fields of view with large responses to cAMP waves. The size of the transcriptional response to cAMP (peak:trough intensity) does not persist between waves (Figure S4D). Together, these observations imply that the transcriptional state is randomized by the cAMP wave. The alternative hypothesis in which stimulation transiently augments transcription before cells return to their previous individual states is not consistent with our observations.

Persistence dictates the timescale over which differences can be perceived between cells. While a measurement of a fixed population provides a snapshot of instantaneous heterogeneity, a short persistence means that the level of transcription quickly fluctuates, and therefore, when single-cell transcription is integrated over time, this heterogeneity is quickly reduced. Transcriptional persistence (also called epigenetic memory) is generally considered to occur over timescales of a cell cycle or greater [30, 31, 32], with chromatin or transcriptional feedback proposed to stabilize expression states [33]. Our data imply that oscillatory transcriptional behavior is a mechanism to rapidly override these slowly varying intrinsic states and, via this consequent reduction in heterogeneity in gene expression, allow accurate cell responses during development. An understanding of the mechanisms by which stochastic responses are integrated to generate a robust transition in space and time is vital in every developmental system, and the combination of approaches we have developed here will allow the timescales of single-cell heterogeneity to be measured quantitatively in the many other contexts in which signal dynamics are crucial in the regulation of gene expression.

## Experimental Procedures

To visualize nascent *csaA* transcripts, we used *Dictyostelium* AX3 cells with 24 MS2 loops inserted into the 5′ UTR of the *csaA* coding sequence [16]. These cells were cotransformed with plasmids expressing RFP-H2B (to facilitate tracking and spot identification) and MS2-GFP [2], and clones expressing both markers were selected using 20 μg/ml G418. Cells grown in HL5 media were prepared for imaging by washing with KK2 (20 mM KPO_4_, pH6.2), plated on KK2/2% agar at 2.5 × 10^6^ cells/cm^2^, and then placed in humidified chambers at 22°C. Prior to imaging, 1 cm squares were excised and inverted onto Bioptechs Delta TPG dishes (0.17 mm). Agar was covered in mineral oil to prevent desiccation. We used a wide-field fluorescence system specifically designed for fast sensitive imaging of photosensitive samples [34]. Four xy positions were imaged for 40 min with 30 s intervals. z stacks consisting of 19 slices with 0.33 μm separation were captured using a GFP/mCherry filter set (Chroma 59022), with 50 ms exposure per slice per channel. UV (Schott GG420) and neutral density filters (Chroma ND0.6A) were used to attenuate illumination. Data were processed to extract cells that were tracked unambiguously for >10 consecutive frames. Image processing is described in the Supplemental Experimental Procedures and Figure S1. For cell mixing, *csaA*MS2 cells were mixed 1:4 with AX3 expressing H2B-mCherry from the genomic *rps30* gene. *csaA*MS2 cells developed 2 or 4 hr were mixed with 4 hr H2B-mCherry cells and replated on agar. Agar was inverted after 1 hr, and streams were imaged after a further 90 min, with four xy positions imaged for each time point of the six experimental repeats.

## Supplemental Information

Document S1. Figures S1–S4, Supplemental Results, and Supplemental Experimental Procedures
