# Supplementary material for: Regulation of Transcriptional Bursting by a Naturally Oscillating Signal
Source: Curr Biol. 2014 Jan 20;24(2):205–11. doi: 10.1016/j.cub.2013.12.011 (PMC3928820; doi:10.1016/j.cub.2013.12.011)
Supplement: Document S1. Figures S1–S4, Supplemental Results, and Supplemental Experimental Procedures [file mmc1.pdf]

Current Biology, Volume *24*

Supplemental Information

Regulation of Transcriptional Bursting by a Naturally Oscillating Signal

Adam M. Corrigan and Jonathan R. Chubb

Figure S1

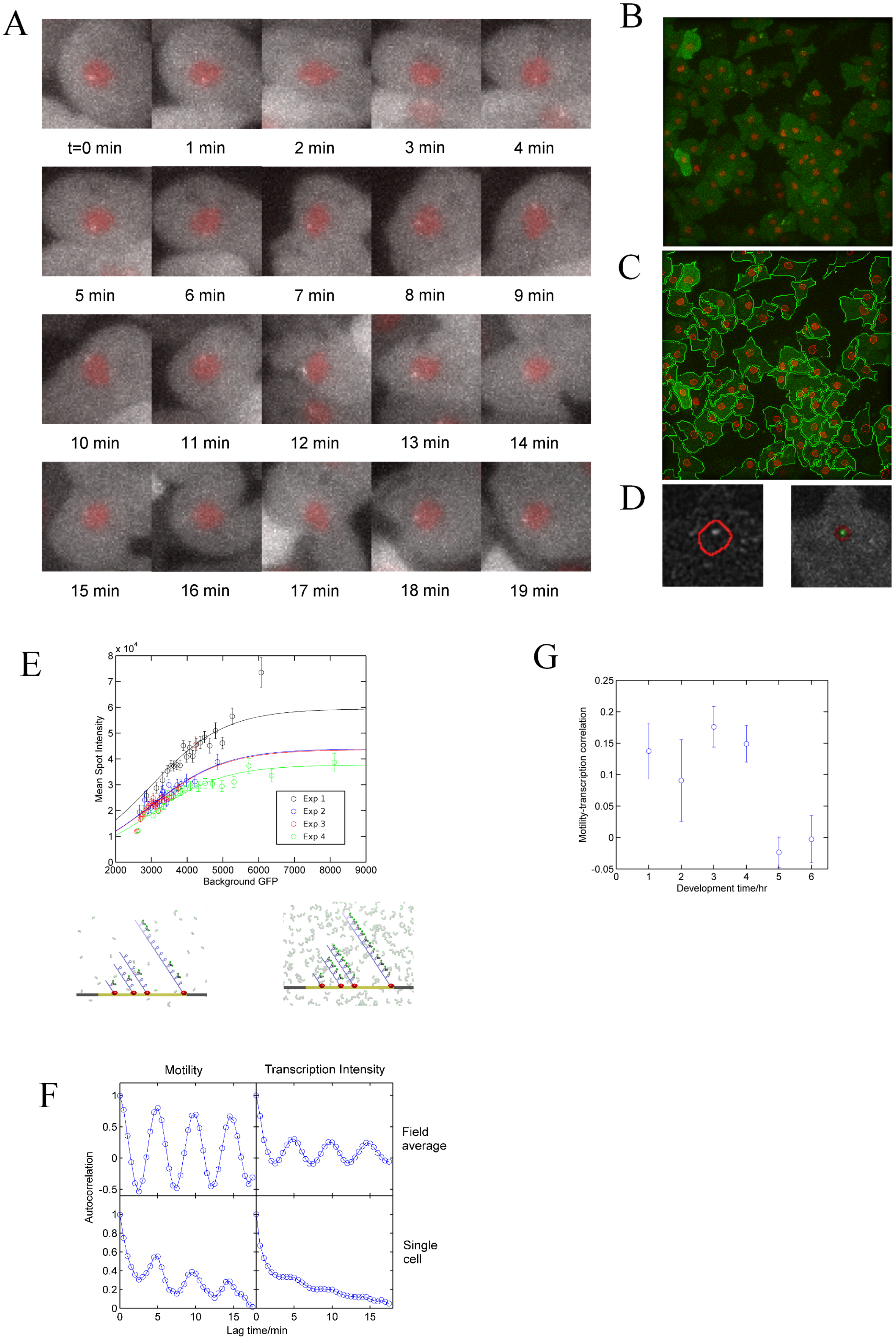

A
t=0 min
1 min
2 min
3 min
4 min
5 min
6 min
7 min
8 min
9 min
10 min
11 min
12 min
13 min
14 min
15 min
16 min
17 min
18 min
19 min
B
C
D
E
x 10^4
Mean Spot Intensity
Background GFP
Exp 1
Exp 2
Exp 3
Exp 4
G
Motility-transcription correlation
Development time/hr
F
Motility
Transcription Intensity
Field average
Single cell
Autocorrelation
Lag time/min

Figure S2

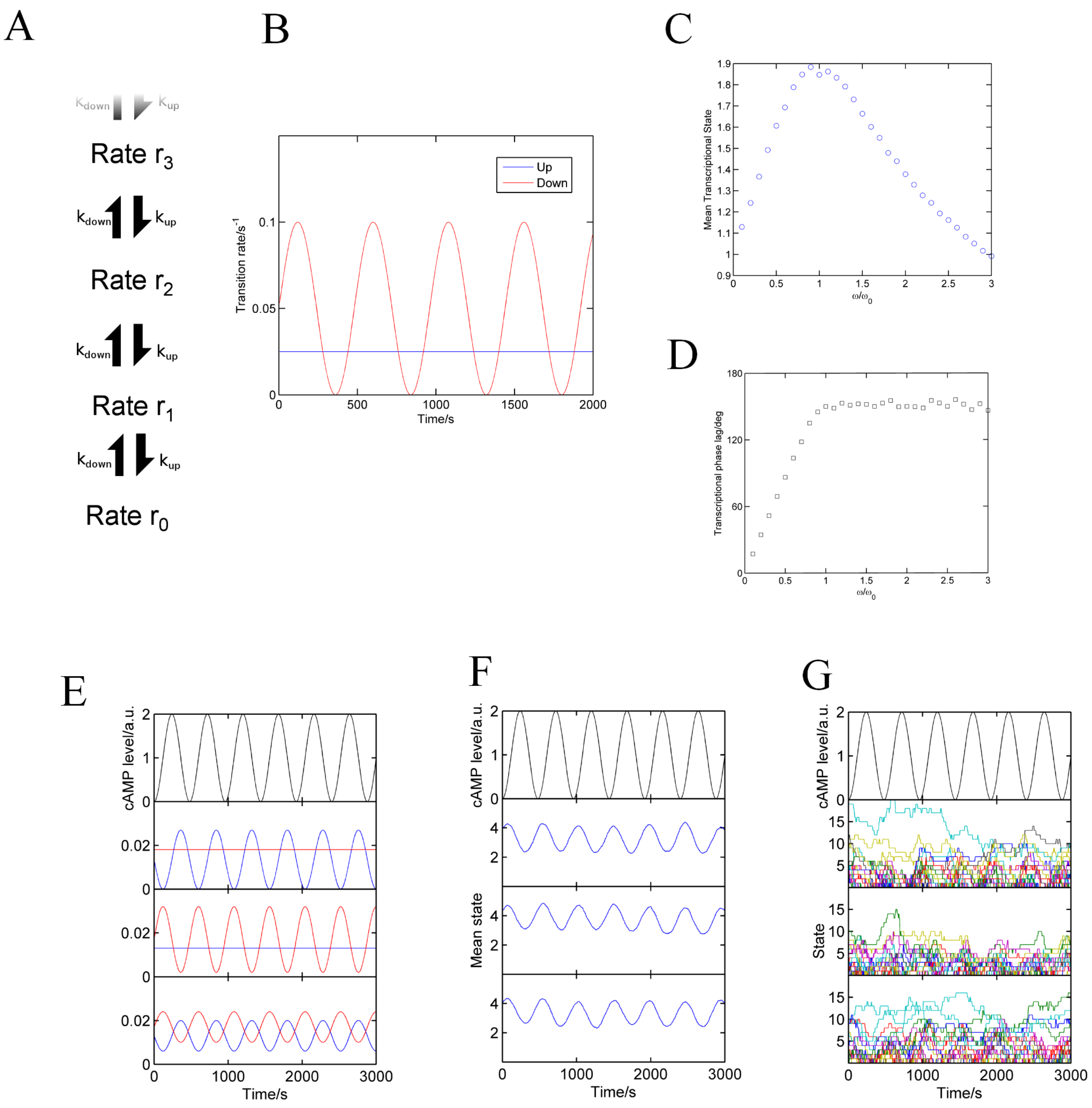
A
B
C
D
E
F
G
Rate r3
Rate r2
Rate r1
Rate r0
kdown
kup
Up
Down
Transition rate/s-1
Time/s
Mean Transcriptional State
ω/ω0
Transcriptional phase lag/deg
cAMP level/a.u.
Mean state
State

Figure S3

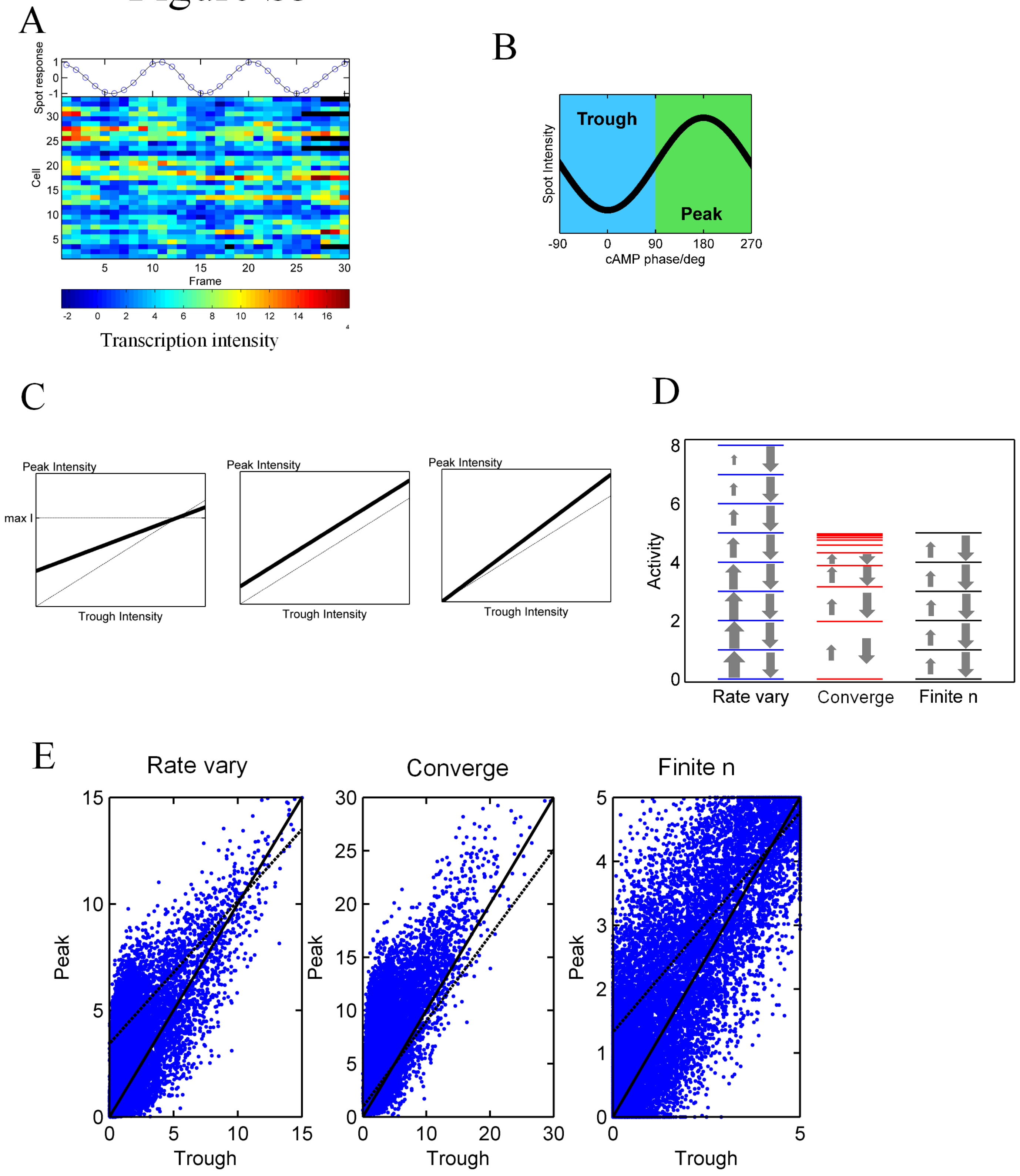

A
Spot response
Cell
Frame
Transcription intensity
B
Trough
Peak
Spot Intensity
cAMP phase/deg
C
Peak Intensity
max I
Trough Intensity
D
Activity
Rate vary
Converge
Finite n
E
Rate vary
Converge
Finite n
Peak
Trough

Figure S4

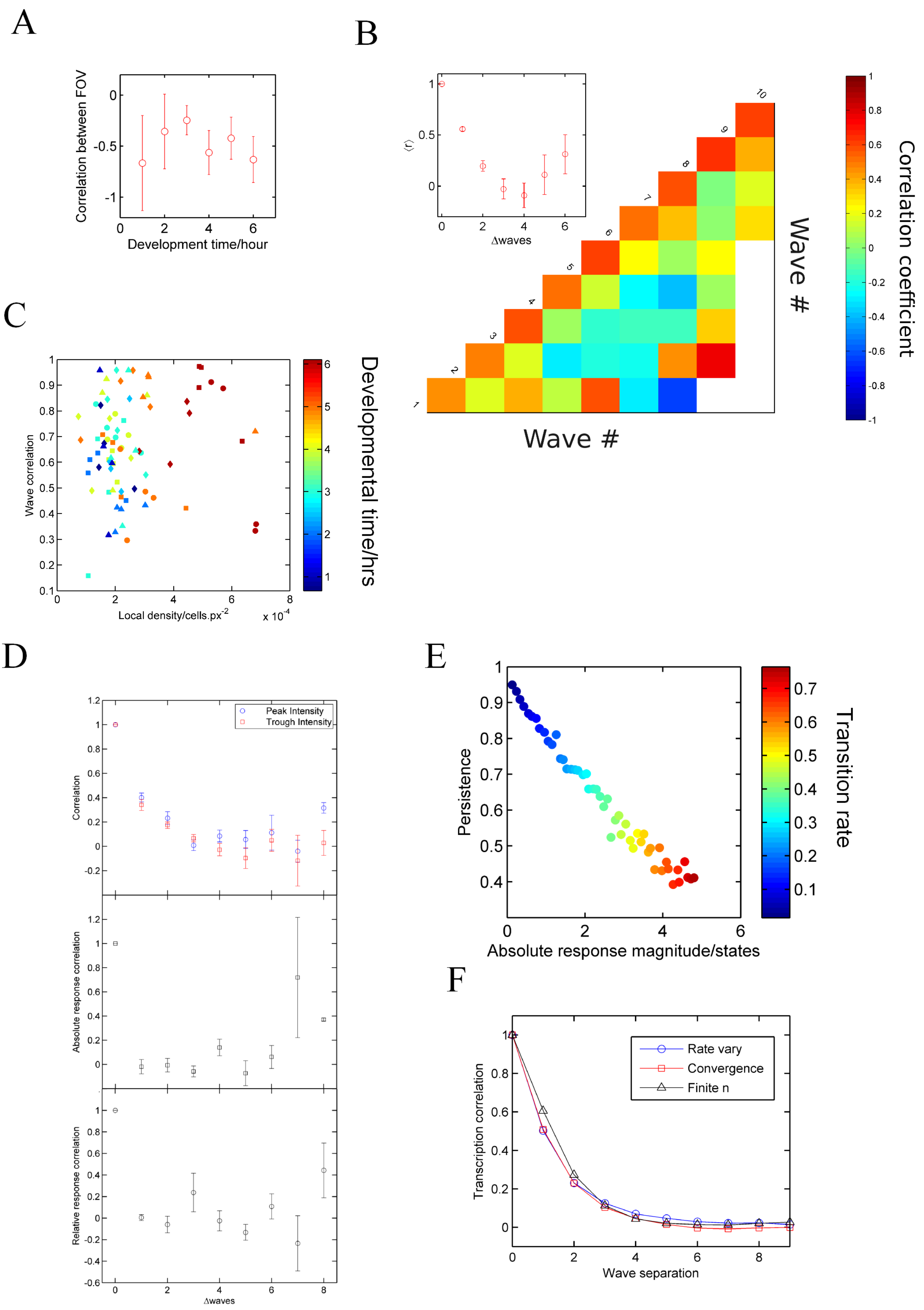
A
B
C
D
E
F
Correlation between FOV
Development time/hour
⟨r⟩
Δwaves
Wave #
Correlation coefficient
Wave correlation
Local density/cells.px^{-2}
x 10^{-4}
Developmental time/hrs
Peak Intensity
Trough Intensity
Correlation
Absolute response correlation
Relative response correlation
Persistence
Absolute response magnitude/states
Transition rate
Rate vary
Convergence
Finite n
Transcription correlation
Wave separation

Supplemental Figure Legends

Figure S1: Image Analysis (relates to Figure 1 and Supplemental Methods)
A) 2D projected stills of a 3D csaA-MS2 movie. Periodic nascent RNA accumulation is observable as fluctuations in nuclear spot intensity. B) Typical two-channel image with the nuclear marker in the RFP channel. 3D image stacks consisting of 19 z-slices in each channel were captured at each time point, for clarity 2D maximum projections are shown. C) Final segmentation result, after marker-based gradient watershed operations on the RFP channel followed by the GFP channel. D) Left: difference-of-Gaussian filtering to detect transcription spots. The nuclear masks are overlaid as red outlines. Right: cylindrical annulus (red) used to calculate spot background. The background is subtracted from spot pixels (green cylinder) used to calculate spot intensity. E) Correcting the spot intensity based on the background GFP level, which determines the occupancy fraction of the nascent MS2 loops. F) Autocorrelations of field of view average properties (top row) and individual cell tracks (bottom row). The properties correlated are two-frame displacement (left column) and transcription intensity (right column). G) Correlation between motility and transcription in single cells, as a function of developmental time. All error bars show S.E.M..

Figure S2: Modelling transcriptional oscillations (relates to Figure 2 and Supplemental Methods)
A) A model of transcriptional activity where transitions occur between states of increasing initiation rate. B) The downward rate constant is chosen to depend on the temporal cAMP gradient, while the upward rate constant is fixed. C) Average transcription state as a function of oscillation frequency. D) Phase lag between transcription and cAMP level as a function of oscillation frequency. E) Different models of *csaA* responsiveness to the cAMP wave, with upward (blue) and downward (red) transition rates dependent on cAMP temporal gradient. Top panel shows cAMP oscillations. 2nd panel shows model varying only the upward transitions (reduced during rising phase and increased during falling phase). 3rd panel shows model varying the downward transitions (the model described in the main text). The 4th panel shows a model allowing both upward and downward rates to vary. F) Fluctuations in mean transcriptional state generated by the three different models in E. G) Fluctuations in single cell transcriptional state generated by the three different models in E.

Figure S3: Response heterogeneity and model architecture (relates to Figure 3 and Supplemental Methods)
A) Enlargement of a representative region of transcription intensity for cells in a field at 5h (lower panel), tracked for up to 4 wave cycles, highlighting the randomness and heterogeneity in transcriptional response compared with the average spot response for the cells shown and sinusoidal fit (upper panel). B) Cartoon of time points used to calculate the trough and peak spot responses to each wave. The solid line indicates the average spot response as in the upper panel of A. C) Cartoons of possible relationships between the trough and peak transcriptional intensities: left – saturation model with gradient inversely proportional to intercept and no response at high trough intensities; centre – uniform response where the peak intensity is greater than the trough intensity by a constant amount; right – proportional model in which the peak intensity increases by a factor proportional to the trough intensity. This is discussed further in the Supplemental Results text. D) Different scenarios for state transitions in the transcriptional oscillation model which account for the saturation in wave response observed experimentally: rate constants may vary such that high activity states have smaller upward rate constants (left), the difference between the transcriptional activities of adjacent states could decrease for higher states (centre) or there may be a finite number of states (right). E) Peak-trough response plots for the three models. Blue dots indicate the mean state in a single trough and subsequent peak, the solid line denotes equal peak and trough intensities, the dotted line is a linear fit to the data.

Figure S4: Cell density and transcriptional persistence as sources of transcriptional heterogeneity (relates to Figure 4 and Supplemental Methods).
A) Average correlation between cell density and spot intensity between fields throughout development. B) Heatmap of correlation coefficients between the mean transcription intensities of distinct waves. Missing elements indicate no cells present in both waves of interest, due to high motility. Inset – correlation averaged over waves of equal separation, showing a characteristic decay in the correlation of response as waves become more widely separated in time. With bigger gaps between waves, correlation values become more noisy. Data are from the 4 fields of view from one of the 5h experimental repeats C) Scatter plot comparing field density and transcriptional persistence (wave correlation). Colour indicates developmental time and different markers denote different experimental days D) Top panel: Persistence of peak and trough transcription intensities, showing a similar trend to the overall transcriptional intensity. There is no persistence in absolute (peak minus trough, (middle) or relative (peak divided by trough, (bottom) responses, suggesting the *response* to each wave is stochastic. E) Model simulation showing fast transition rates have high responses and short persistence, while systems with slow transition rates have small responses and correspondingly long persistences. F) Model simulations of transcriptional persistence with different types of state transition ladder (relates to Figure S3C). E and F are discussed further in the Supplemental Results text. All error bars show S.E.M..

Supplemental Results

Considerations for models of csaA transcription

The measurements we have made place a number of constraints or requirements for any model describing the phenomenon. Firstly, and as described in the main text, as the waves become faster between 4 and 6 hours of development transcription remains approximately anti-phase with the motility cycle, ruling out a simple time delay as the sole regulator of the transcription lag. We therefore propose that transcription is enhanced during one phase of the cAMP wave and suppressed in another, tuning the duration of the response in proportion to the wave period. A simple method of achieving this differential induction is by linking the gene activity to the cAMP dynamics, either the absolute level, or the temporal or spatial gradient. We model the activity of the csaA gene as having a number of discrete states of monotonically increasing polymerase initiation rate, with the rate constants for transitions between states depending on the temporal gradient of cAMP level, such that downward transitions are enhanced during the rising phase of the cAMP wave and suppressed during the falling phase, while holding the rate of upward transitions constant. Alternative models from which the same antiphase effect can be achieved are by varying only the upward transitions (reduced during rising phase and increased during falling phase) or by allowing both upward and downward rates to vary (panels in figure S2E); the phase behaviour is produced by modulating the ratio of upward to downward rates in a cAMP-phase-dependent manner. The phase behaviour of the average state (Figure S2F) and the high level of heterogeneity at the single cell level (Figure S2G) is maintained for each class of model. We chose only downward rates varying to keep the model as simple as possible, and also because our local density results suggest that high cAMP levels may have a suppressive effect on transcription. The rates may not depend directly on the extracellular cAMP gradient, rather the concentration, state or localisation of some intermediate signalling molecule which is linked to cAMP dynamics.

The observation that spot intensity decreased by 6 hours of development, when cAMP waves displayed shorter periods of 4 minutes or less, led us to ask if the frequency of stimulation could account for the level of transcriptional output. One method of producing this effect relies on the finite time required for a population of cells to move from the rising-phase, low activity state to the falling-phase high activity state. If rate constants are sufficiently slow such that the system does not reach equilibrium (ensured by setting the downward rates to be greater than the upward rates) during the falling phase of the wave, then faster waves will act to suppress transcription before the maximum level is reached. An alternative model for the dampening effect is to include some activating species which is depleted and is incompletely replenished at high wave frequencies. Since this introduces a number of additional free parameters, we continue to use the considerably simpler model capturing the frequency and phase response.

As development proceeds, the gradient and absolute level of extracellular cAMP will both increase, as well as the frequency of oscillation, and cell mixing experiments also reveal a developmental time effect on transcriptional state. The single-cell wave responses and the transient transcriptional persistence both have implications for the class of model used to describe the system. Experimentally the response gradient is always measured to be less than 1, and from this we infer that the initiation rate is the most likely candidate for regulation by the cAMP signal. The implication that transcription sites with a large amount of nascent RNA tend to be affected by cAMP to a lesser degree in absolute terms than sites with less or no detectible RNA suggests that the initiation rate is less strongly influenced if the transcriptional activity is already high (Figure S3C, left). There are alternative models for the transcriptional response, including a constant increase in spot intensity independent of trough behaviour (Figure S3C, centre) or an increase in the polymerase dwell time by delaying RNA cleavage (Figure S3C, right). These models, however, are not consistent with the experimental observations of a strong inverse relationship between the response gradient and the response intercept (Figure 3C) and a response gradient less than one. The

constant increase model would have a response gradient of 1 and a non-zero intercept independent of the gradient (Figure S3C, centre). The alternative model of increasing the transcription intensity by lengthening the RNA cleavage time predicts that the absolute increase in intensity is proportional to the pre-existing amount of transcriptional activity, giving an intercept of zero and a gradient larger than 1 (FigureS3C, right).

By measuring the trough spot intensity and corresponding peak intensity we found that cells have a maximum level of transcription, above which there is no response to the cAMP wave. In terms of the ladder of transcriptionally active states, this observation can be interpreted in several ways. Firstly, the rate constants may vary such that high activity states have smaller upward rate constants (Figure S3D, left), hence decreasing the probability of a cell in a high state moving further upwards. Similarly, the difference between the transcriptional activities of adjacent states could decrease for higher states such that the states converge on a finite maximal activity (Figure S3D, centre). Finally, there may be a finite number of states (Figure S3D, right). Conceptually, there is little difference between these models, effectively defined by the state-dependent rate constants, and each can accurately describe the peak-trough response (Figure S3E).

The transient persistence observed means that each cAMP wave does not fully determine the transcription state of a cell. In other words, the rate constants for state transitions must be sufficiently slow that the state is randomized only after 20 minutes or longer, while being fast enough to give a robust single-cell response to the stimulus. In our model the response to a wave is not transient; after peaking each cell does not return to its previous trough level, but behaves stochastically. The effect of this is to link the level of response and duration of persistence such that systems with fast transition rates have high responses and short persistence, while a system with slow transition rates has a small response and correspondingly long persistence (Figure S4E) as observed experimentally. The absolute values for rate constants depend on the number of states in the model, since the persistence timescale is effectively determined by the time required to move from a high activity to a low activity state. The three classes of model described in figure S3D and E have the rate constants globally scaled to give persistence times comparable with the experimental system (Figure S4F). Currently, the system is underdetermined to the extent that absolute parameters cannot be estimated. Nevertheless, we have identified a number of requirements that a model must satisfy in order to adequately describe the system.

Supplemental Experimental Procedures

Automated Image Processing and Analysis

Nuclei, cells and transcription spots were identified and segmented using custom software written in MATLAB (Mathworks). The software is available from the authors on request. Frames were considered individually, and tracking performed after cell identification. Cells with nuclear markers were used (Figure S1A and S1B), as prior identification of nuclei greatly improved cell identification and segmentation. Methods described here are generally applicable in 3D, however for speed in processing many frames, and since overlapping cells were rare, images were segmented first in 2D using maximal projections then in z.

For nuclear segmentation, 2D maximum projections of nuclei images were blurred with a Gaussian kernel. A local maxima filter found the rough centre of each nucleus. A customized gradient watershed algorithm defined 2D nuclei boundaries. To obtain 3D information, each column of pixels was considered in turn; the points of steepest gradient either side of the maximum give the top and bottom of the nucleus. The 3D volume was morphologically smoothed to remove effects of noisy pixels. Cell volume was segmented similarly, using nuclear locations as foreground markers for the watershed process. A typical segmentation result is displayed in Figure S1C.

Transcription spots were visualized as bright objects of characteristic size in the GFP channel. A 3D difference-of-Gaussians filter enhanced objects within the correct size range (Figure S1D, left). By capturing closely spaced z-slices, transcription spots were present in multiple adjacent slices, a condition used to reduce noise effects. The nuclear volume was used to find the pixel with the highest response to the spot filter within each nucleus. Spot intensity was calculated by summing intensity within a cylinder around the measured spot position. Cell background intensity was calculated by averaging a cylindrical annulus around the spot, and subtracted from each spot pixel. Cylinder sizes are shown in Figure S1D (right). Use of a nuclear marker prevented mis-assignment of cytoplasmic autofluorescence.

Cell positions were determined independently for each frame. To measure dynamic properties over time, cells were tracked between frames using a custom algorithm to minimize the global sum of squared displacements (SSD) between frames. The likelihood of a match between a cell in one frame and a cell in the next was calculated from the total SSD of configurations which contained the match. High SSDs were given a low weighting, and low SSDs a high weighting. Thus the global effect of a cell-cell match was taken into consideration, and configurations other than the outright minimum SSD were allowed to contribute, to assess the level of any tracking uncertainties. There is significant variegation in the cytoplasmic intensities of the MS2-GFP and H2B-RFP from cell to cell and this was used to aid tracking; changes in intensity were added to changes in x and y in the SSD. Weights for fluorescence and displacement contributions were determined empirically. The accuracy of the tracking algorithm was optimised at multiple developmental time points with manual verification.

Correcting for Background MS2-GFP level

We have observed a cell-to-cell variation in the expression level of the MS2-GFP fusion protein. Spot intensities in low background cells are lower than those in high background cells. This effect has been noted previously [S1], and results from incomplete occupancy of the RNA stem loops by the MS2 protein. Therefore an equal quantity of stem loop RNA in a low background cell will have fewer MS2 dimers bound, and thus a lower spot intensity than in a high background cell. Since we wish our spot measurements to reflect the level of nascent RNA, spot intensities must be corrected by a factor dependent on the background GFP level. To achieve this, spot intensity measurements are grouped into bins of 50 cell tracks based on the background GFP intensity. For each distribution, a scaling factor is calculated so as to shift the distribution to overlap with a reference distribution, chosen to be the central bin. Scaling the distributions so that they superpose carries the assumption that the underlying distribution of stem loop numbers is independent of background GFP level. This procedure was carried out for spots of the *act5* and *csaA* genes, and the scaling factor

was found to be independent of the gene of interest, instead reflecting the binding behaviour of the MS2-GFP protein (Figure S1E). The scaling factor data points were then fit with a smooth function, allowing the correction of spots in cells of arbitrary background intensity. The corrected spot intensity was found to be uncorrelated with background intensity using the Pearson product-moment correlation coefficient.

Wavelet analysis

Wavelets are increasingly used for analysis of temporally varying data, particularly in systems oscillating in a non-robust manner where the frequency can vary over time, and where one is interested in temporal variations as well as frequency behaviour. To determine phase behaviour, we used a wavelet analysis capable of measuring the amplitude and phase of the motility waves in a manner which is unbiased and robust to noise [S2]. To identify the frequency component of the signal with the highest amplitude we used a family of generalized Morse wavelets, using a toolbox of functions [S3]. We took the motility over time (solid line, Figure 1D upper) and calculated the wavelet transform, oversampling in frequency space (heatmap, Figure 1D lower), then used an algorithm to identify ridge points in the transform taking the ridge with the maximum amplitude for each frame. From this the phase of the motility wave was extracted (background colour, Fig. 1D upper). The link between cAMP and cell motility is well established, although in dense fields of view the physical constraints of other cells in close proximity may prevent cells responding to the timing and direction of the cAMP gradient with maximum fidelity. Therefore, when waves of motility were robust, since no systematic variation in timing is observed across fields of view, the phase was averaged over each cell in the field to provide the best estimate of cAMP behaviour. At earlier times, before synchronous waves are robustly set up, the phase of each cell was used.

Circular statistics

Because the phase is cyclic, that is 0° is equivalent to 360°, special methods must be employed to calculate the mean and standard deviation [S4]. The mean angle $\langle\theta\rangle$ is calculated using:

$$R e^{i\langle\theta\rangle} = \frac{1}{N}\sum_j e^{i\theta_j} \qquad \text{(S1)}$$

The circular standard deviation is given by:

$$\sigma_0 = \sqrt{-2\ln R}, \qquad \text{(S2)}$$

where R is defined in equation S1 (above). The standard error in the mean is calculated as:

$$\sigma_m = \frac{\sigma_0}{\sqrt{N}}. \qquad \text{(S3)}$$

Combining Errors in the Correlation Coefficient

Correlation matrices have a large number of elements. We are primarily interested in how the wave separation affects the correlation in response, and this required averaging the elements along the diagonals. Using Fisher's z-transformation for the product-moment correlation coefficient:

$$z' = \frac{1}{2}\left[\ln(1+r) - \ln(1-r)\right], \qquad \text{(S4)}$$

produces a variable which is approximately normally distributed, and the error in the coefficient is given by:

$$\sigma_r = \frac{1 - r^2}{\sqrt{N - 3}} \qquad \text{(S5)}$$

where N is the number of data points used to calculate the correlation in each case. Elements which have fewer than 5 cells contributing to them (due to cells entering and leaving the field of view between early and late waves) were removed from the average due to the size of the errors being systematically underestimated. A weighted average is then calculated, with the weight for each element given by $1/\sigma^2$.

Measurement of cell parameters
The 2-frame displacement was used as a sensitive measure of chemotactic response as it reduces noise and provides a good estimate of instantaneous motion at frame capture. Two-frame displacement was defined for frame, *t*, as the distance between the previous frame (*t*-1) and the next (*t*+1). The local density of cells around each cell in each frame was calculated by counting the number of cells lying within a circle of a chosen radius, and dividing by the area of the circle (bounded by the field of view). A radius of 75 pixels (18 μm) was chosen to include more than only immediately adjacent cells but retain variation within a field of view. Quantitatively similar behaviour was observed for radii of 50 or 100 pixels.

Supplemental References

S1. Wu, B., Chao, J.A., and Singer, R.H. (2012). Fluorescence fluctuation spectroscopy enables quantitative imaging of single mRNAs in living cells. Biophysical journal *102*, 2936-2944.

S2. Lilly, J.M., and Olhede, S.C. (2012). Generalized Morse Wavelets as a Superfamily of Analytic Wavelets. Ieee T Signal Proces *60*, 6036-6041.

S3. Lilly, J.M. (2012). JLAB: Matlab freeware for data analysis. 0.94 Edition. (http://www.jmlilly.net/software.html).

S4. Berens (2009). CircStat: A MATLAB Toolbox for Circular Statistics. Journal of Statistical Software *31*, 1.
